# Sex-differential genetic effect of phosphodiesterase 4D (PDE4D) on carotid atherosclerosis

**DOI:** 10.1186/1471-2350-11-93

**Published:** 2010-06-12

**Authors:** Yi-Chu Liao, Hsiu-Fen Lin, Yuh-Cherng Guo, Ming-Lung Yu, Ching-Kuan Liu, Suh-Hang Hank Juo

**Affiliations:** 1 Graduate Institute of Medicine, Kaohsiung Medical University, No. 100, TzYou First Road, Kaohsiung, 80708, Taiwan; 2 Department of Neurology, Kaohsiung Medical University, No. 100, TzYou First Road, Kaohsiung, 80708, Taiwan; 3 Department of Medical Genetics, Kaohsiung Medical University, No. 100, TzYou First Road, Kaohsiung, 80708, Taiwan; 4 Department of Medical Research, Kaohsiung Medical University Hospital, No. 100, TzYou First Road, Kaohsiung, 80708, Taiwan; 5 Section of Neurology, Taichung Veterans General Hospital, No. 160, Sec. 3, ChungKang Rd., Taichung, 40705, Taiwan; 6 Department of Neurology, Kaohsiung Medical University Hospital, No.100, ShihChuan 1st Road, Kaohsiung, 80708, Taiwan; 7 Department of Neurology, Kaohsiung Municipal Hsiao-Kang Hospital, No.482, Shanming Road, Kaohsiung, 81267, Taiwan; 8 Department of Internal Medicine, Kaohsiung Municipal Ta-Tung Hospital, Kaohsiung Medical University Hospital, Kaohsiung Medical University, No. 100, TzYou First Road, Kaohsiung, 80708, Taiwan; 9 Department of Neurology, National Yang-Ming University, No.155, Sec.2, Linong Street, Taipei, 11221, Taiwan

## Abstract

**Background:**

The phosphodiesterase 4D (PDE4D) gene was reported as a susceptibility gene to stroke. The genetic effect might be attributed to its role in modulating the atherogenic process in the carotid arteries. Using carotid intima-media thickness (IMT) and plaque index as phenotypes, the present study sought to determine the influence of this gene on subclinical atherosclerosis.

**Methods:**

Carotid ultrasonography was performed on 1013 stroke-free subjects who participated in the health screening programs (age 52.6 ± 12.2; 47.6% men). Genotype distribution was compared among the high-risk (plaque index ≥ 4), low-risk (index = 1-3), and reference (index = 0) groups. We analyzed continuous IMT data and further dichotomized IMT data using mean plus one standard deviation as the cutoff level. Because the plaque prevalence and IMT values displayed a notable difference between men and women, we carried out sex-specific analyses in addition to analyzing the overall data. Rs702553 at the PDE4D gene was selected because it conferred a risk for young stroke in our previous report. Previous young stroke data (190 cases and 211 controls) with an additional 532 control subjects without ultrasonic data were shown as a cross-validation for the genetic effect.

**Results:**

In the overall analyses, the rare homozygote of rs702553 led to an OR of 3.1 (p = 0.034) for a plaque index ≥ 4. When subjects were stratified by sex, the genetic effect was only evident in men but not in women. Comparing male subjects with plaque index ≥ 4 and those with plaque index = 0, the TT genotype was over-represented (27.6% vs. 13.4%, p = 0.008). For dichotomized IMT data in men, the TT genotype had an OR of 2.1 (p = 0.032) for a thicker IMT at the common carotid artery compared with the (AA + AT) genotypes. In women, neither IMT nor plaque index was associated with rs702553. Similarly, SNP rs702553 was only significant in young stroke men (OR = 1.8, p = 0.025) but not in women (p = 0.27).

**Conclusions:**

The present study demonstrates a sex-differential effect of PDE4D on IMT, plaque index and stroke, which highlights its influence on various aspects of atherogenesis.

## Background

Stroke is a heterogeneous multifactorial disorder, to which both the environmental and genetic factors contribute [1,2]. The phosphodiesterase 4D (PDE4D) gene was identified as a stroke susceptibility [3]. Several studies tried to replicate this association in different populations but the results were conflicting [4-18]. The complex entity and heterogeneity of stroke might be responsible for the inconsistent findings among studies. Given that atherosclerosis proceeds long before the occurrence of clinical ischemic event, using the severity of carotid atherosclerosis as a phenotype may reduce the complexity and improve the power to detect the PDE4D genetic effect.

Carotid intima-media thickness (IMT) and plaques have been shown to be good independent predictors for future vascular events [19,20]. Despite the fact that both phenotypes correlate well to the pathologically and clinically defined atherosclerosis, they represent distinct traits with unique determinants and relationships to atherosclerosis [21]. IMT is akin to a physical effect adapting to aging and hypertensive stress whereas plaque corresponds to a more pathogenic alternation in the vessel walls [22]. Therefore, these two phenotypes can be used as independent surrogate markers in the genetic studies of atherosclerosis.

Sex-specific relationships between IMT and the environmental factors like triglyceride, smoking and physical activities have been demonstrated in the Tromsφ study [23]. In addition, the association between inflammatory markers and IMT progression is stronger in women than in men [24]. The lower incidence of cardiovascular disease in premenopausal women [25] suggests a different susceptibility to atherosclerosis in men and women. Accordingly, the genetic effect on IMT or plaque may be modified by sex.

Among the literature investigating the associations between the PDE4D gene and stroke, only one study included IMT and plaque as phenotypes of interest [26]. This study only enrolled participants between ages 50 and 65; thus their results may not generalize to the population at large. In the present study, we sought to determine whether the PDE4D gene is involved in the pre-clinical atherogenic process, ranging from the early changes in the vessel morphology (i.e. IMT) to a later stage of atherosclerosis (i.e. plaque formation). The SNP rs702553 at the PDE4D gene (i.e. SNP56 from the original study by Gretarsdottir et al.[3]) was selected because it conferred a risk to young ischemic stroke in our previous study [27]. To clarify the influence of sex on the relationship between the PDE4D gene and carotid atherosclerosis, we first performed the analyses in the overall data and then analyzed the data from men and women separately.

## Methods

### Subjects

The study subjects were stroke- and myocardial infarction (MI)-free volunteers who participated in the health screening programs at the Kaohsiung Medical University Hospital between January 2006 and December 2007. A total of 1013 subjects were enrolled for carotid ultrasonography examinations. To compare the genetic effect across the atherosclerotic phenotypes, we also used the previous young stroke data (including 190 cases and 211 age-matched controls) [27] with an additional 532 control subjects who were younger than 45 years old. These young subjects were recruited from the Kaohsiung Medical University and did not receive carotid ultrasound examinations. All of the study participants were descendants of Han Chinese resided at southern Taiwan.

Demographic data and histories of hypertension, diabetes mellitus, and cigarette smoking were obtained from each study subject. Blood pressure measurements were done using a calibrated standard aneroid sphygmomanometer (Omron; Vernon Hills, Illinois) after sitting for at least 5 minutes and the average values from two measurements were used. A subject was defined as having hypertension if he had systolic blood pressure ≥140 mm Hg or diastolic pressure ≥90 mm Hg, or was taking anti-hypertensive medications. A subject was defined as having diabetes if he had fasting blood glucose ≥126 mg/dl or was taking hypoglycemic medications. A subject was defined as having hyperlipidemia if he had serum levels of total cholesterol (TC) ≥ 240 mg/dl. Overnight fasting venous blood was collected for biochemistry analyses and genetic studies. Serum levels of TC were determined using standardized enzymatic procedures (Boehringer Mannheim, Germany). A subject was defined as a smoker if he ever smoked (including current or past smoker). All study protocols and methods were approved by the local Institutional Review Board (IRB).

### Carotid ultrasonography studies

The ultrasonic examinations were assessed using the Philips HD 11 ultrasonography system equipped with a 7.5 Hz to 10 Hz linear array transducer (Philips Medical Systems, Bothell, Washington, US). An experienced technician who was blinded to the patients' clinical data performed all the ultrasonic measurements. Subjects were examined in supine position. The transducer scanning direction was using anterior-oblique insonation. The far walls of carotid IMT were visualized bilaterally and the IMT values were measured at the plaque-free area of the common carotid artery (CCA, 10 to 20 mm proximal to the tip of the flow divider), carotid bifurcation (Bif, tip of the flow divider and extending 10 mm proximally) and internal carotid artery (ICA, proximal 10 mm above the bulb) separately. We used an automated computerized analyzing system (Philips Qlab quantification software) to improve the measurement accuracy. This new computerized technique automatically detected the echo interfaces [28] and manual corrections were performed when there was no automatic outlining of the lumen-intima or the media-adventia interfaces. All the ultrasonic data were interpreted by a single neurologist (HF Lin). The mean absolute difference and standard deviation (SD) between two measurements was 0.05 ± 0.04 mm.

Carotid plaque, defined as an area of focal protrusion into the lumen at least 50% greater than the surrounding wall thickness, was measured in 682 out of the 1013 participants. The degree of plaque was graded using the following criteria [29]: grade 0, no observable plaque; grade 1, one small plaque (<30% of the vessel diameter); grade 2, one medium plaque (30-50% of the vessel diameter) or multiple small plaques; grade 3, one large plaque (>50% of the vessel diameter) or multiple plaques with a least one medium plaque. The plaque index was calculated by the summation of plaque grades from five segments of the carotid arteries bilaterally (proximal and distal CCA, Bif, ICA and extra-cranial carotid artery), which was a modified method based on the Sutton-Tyrrell's study [29].

### Genotyping

Genomic DNA was isolated from the whole blood using Puregene kit according to the manufacturer's protocols (Gentra, Research Triangle, NC). Previously, we screened four single nucleotide polymorphisms (SNPs) that were implicated to be associated with stroke in the literature. The four SNPs were rs12188950 (SNP45), rs702553 (SNP56), rs966221 (SNP83), and rs2910829 (SNP87). We found that the SNP rs702553 was significantly associated with young ischemic stroke in the Taiwanese population [27]. In the present study, we further tested whether this SNP was also related to other atherosclerotic phenotypes. Genotyping was performed by the Applied Biosystems TaqMan technology. Briefly, PCR primers and two allelic-specific probes were designed to detect the specific SNP target. The PCR reactions were performed in the 96-well microplates with an ABI 7500 real-time PCR machine (Applied Biosystems, Foster City, USA). Allele discrimination was achieved by detecting fluorescence using its System SDS software version 1.2.3. The genotype calling rate was 97.1%.

### Statistical analysis

Genotype distribution was tested for Hardy-Weinberg equilibrium (HWE) using the Goodness-of-fit test. Two phenotypes (IMT and plaque index) were used as proxies for carotid atherosclerosis. For carotid plaque, those with a plaque index ≥ four were defined as high-risk individuals, subjects with an index between one and three were defined as low-risk individuals, and those without any observable plaque (index = 0) were defined as reference individuals. Conditional logistic regression and the chi-squared test were used to evaluate the difference in genotype distribution.

We analyzed both the continuous IMT data as well as the dichotomized IMT data. For the dichotomized IMT data, subjects with IMT values above the sex-specific and age-adjusted mean plus one SD were defined as high-risk individuals, and the rest of study subjects were defined as reference individuals. Logistic regression with adjustment for other cardiovascular risk factors (diabetes, hypertension, hyperlipidemia and smoking) were used to estimate the odds ratio (OR) and 95% confidence interval (CI) for the risk genotype. For the continuous IMT data, age-adjusted IMT values were log-transformed to approximate the normal distribution. ANOVA and Student's t-test were then used to compare the mean IMT values across different genotypes.

We analyzed the genetic effect on the overall data as well as the sex- specific genetic effect by stratifying the subjects into men and women. The sex-genotype interaction was further evaluated by (1) adding an interaction term into the regression model, and (2) comparing the difference of odds ratios in men and women using Woolf's logit method and Z-test [30]. Since we tested the PDE4D effect on seven phenotypes (i.e. plaque index, dichotomized IMT data at three segments, continuous IMT values at three segments) and performed the overall and sex-stratification analyses (i.e. a total of 21 tests in Table three), Tukey's ad hoc correction was adopted for multiple testing correction [31]. To evaluate the genetic effect on stroke, logistic regression was used to estimate the OR for each genotype. We also performed subgroup analyses stratified by sex. All statistical analyses were performed with SPSS statistical software (version 13.0).

## Results

The demographic features of the study population are shown in Table 1. The genotype distribution was in HWE. The minor allele frequency of rs702553 in the present study was 0.41, which was quite close to those reported in the three independent Japanese populations (0.42-0.46) [6].

**Table 1:** Demographic characteristics of study subjects

	Young stroke study		IMT study
	
	case (N = 190)	control (N = 743)	overall (N = 1013)
Age(yr)	38.1 ± 6.7 (15-45) †	26.0 ± 8.1 (16-45)	52.6 ± 12.2 (17-87)
Male	124(65.3%)†	336(45.2%)	482(47.6%)
Diabetes	65(34.2%) †	8(1.1%)	103(10.2%)
Hypertension	76(42.5%)†	45(6.1%)	318(31.4%)
Hyperlipidemia	18(10.8%)†	22(3.1%)	53(5.4%)
Smoker (current + past vs. never)	89(48.1%)†	60(8.1%)	208(20.7%)
Total cholesterol (mmol/L)	4.77 ± 1.30†	4.36 ± 0.89	4.83 ± 0.87
PDE4D rs702553			
AA	60(32.6%)	278(37.9%)	352(36.3%)
AT	80(43.5%)	339(46.2%)	451(46.4%)
TT	44(23.9%) *	117(15.9%)	168(17.3%)

* p < 0.05, † p < 0.001 compared with controls of young stroke study.

Quantitative data are presented as mean ± SD

Among the 1013 subjects with IMT data, men had significantly higher IMT values than women at any of the three carotid segments (p = 7.7 × 10^-9 ^- 1.8 × 10^-16^; Table 2). The average age-adjusted IMT values (mm) in men and women were 0.64 vs. 0.59 at CCA, 0.67 vs. 0.63 at Bif, and 0.54 vs. 0.48 at ICA. The cutoff levels (mm) to define the high-risk and low-risk groups at CCA, Bif and ICA were 0.77, 0.80 and 0.64 respectively in men; and 0.69, 0.73, and 0.57 respectively in women. Among the total study participants, data on plaque index was available for 682 subjects. Up to 10.8% of the male subjects had a plaque index ≥ four; whereas 1.7% of the female subjects had a plaque index ≥ four (p = 1 × 10^-7^, Table 2). There was a moderate association between the plaque index and the age-adjusted IMT value from each carotid segment (Pearson's correlation coefficient (r) = 0.24-0.35, p < 0.001).

**Table 2:** Sex-differential distribution among carotid IMT, plaque index and cardiovascular risk factors (only subjects with ultrasonic data are presented)

	Men (N = 482)	Women (N = 531)	p value*
Age(yr)	52.5 ± 12.2	52.8 ± 12.2	0.766
Diabetes	60(12.5%)	43(8.2%)	0.025
Hypertension	171(35.5%)	147(27.7%)	0.008
Hyperlipidemia	24(5.1%)	29(5.6%)	0.704
Smoking (current + past vs. never)	198(41.4%)	10(1.9%)	9.9 × 10^-54^
Total cholesterol (mmol/L)	4.79 ± 0.87	4.86 ± 0.87	0.180
Carotid plaque	N = 268	N = 414	
No plaque (index = 0)	159(59.3%)	288(69.6%)	ref.
Low-risk group (index = 1 - 3)	80(29.9%)	119(28.7%)	0.260
High-risk group (index ≥ 4)	29(10.8%)	7(1.7%)	1 × 10^-7^
Age-adjusted carotid IMT (mm)	N = 482	N = 531	
CCA IMT	0.64 ± 0.13	0.59 ± 0.10	4.2 × 10^-9^
Bif IMT	0.67 ± 0.13	0.63 ± 0.10	7.7 × 10^-9^
ICA IMT	0.54 ± 0.11	0.48 ± 0.08	1.8 × 10^-16^

* Student's t-test or Chi-squared test.

Quantitative data are presented as mean ± SD

The prevalence of the cardiovascular risk factors (hypertension, diabetes, and smoking) was much higher in men than in women (all p values < 0.05, Table 2). Sex remained as an independent determinant of IMT values in the multivariate regression no matter which carotid segment was analyzed (p = 0.001 - 5.5 × 10^-12^).

Three genetic models (assuming the rare allele having a dominant, recessive, or additive effect) were used to evaluate the associations between rs702553 and the atherosclerotic phenotypes. The rare allele appeared to exert a recessive effect and the TT genotype was associated with increased IMT values and a higher plaque index (Table 3). Using individuals with no plaque as the reference group, the TT genotype was over-represented in individuals with a plaque index ≥ four (OR = 3.1, nominal p = 0.034, corrected p = 0.15). Actually, the TT genotype was only significantly related to the high plaque index (plaque index ≥ four) in men (OR = 5.9, nominal p = 0.008, corrected p = 0.036) but not in women. There was no significant association between the SNP rs702553 and the low-risk group (plaque index = 1-3).

**Table 3:** The association between rs702553 at the PDE4D gene and carotid atherosclerosis

Atherosclerotic risk	Overall			Men			Women		
	
	AA + AT	TT	statistics	AA + AT	TT	statistics	AA + AT	TT	statistics
Carotid plaque	N = 544	N = 108		N = 216	N = 40		N = 328	N = 68	
No plaque (index = 0)	354 (83.7%)	69 (16.3%)	ref.	129 (86.6%)	20 (13.4%)	ref.	225 (82.1%)	49 (17.9%)	ref.
Low-risk (index = 1-3)	163 (84.5%)	30 (15.5%)	OR = 1.0 (0.6-1.7) p = 0.934	66 (84.6%)	12 (15.4%)	OR = 1.5 (0.7-3.6) p = 0.312	97 (84.3%)	18 (15.7%)	OR = 0.8 (0.4-1.5) P = 0.534
High-risk (index ≥ 4)	27 (75.0%)	9 (25.0%)	OR = 3.1 (1.1-8.7) p = 0.034	21 (72.4%)	8 (27.6%)	OR = 5.9 (1.6-21.5) p = 0.008	6 (85.7%)	1 (14.3%)	OR = NA

Age-adjusted IMT(mm)	N = 775	N = 162		N = 367	N = 76		N = 408	N = 86	
CCA	0.61 ± 0.12	0.62 ± 0.11	p = 0.150	0.63 ± 0.14	0.66 ± 0.13	p = 0.041	0.59 ± 0.10	0.59 ± 0.08	p = 0.862
Bif	0.65 ± 0.12	0.66 ± 0.13	p = 0. 396	0.67 ± 0.12	0.70 ± 0.14	p = 0.068	0.63 ± 0.11	0.62 ± 0.09	p = 0.472
ICA	0.51 ± 0.10	0.51 ± 0.11	p = 0.518	0.53 ± 0.11	0.55 ± 0.13	p = 0.111	0.48 ± 0.08	0.48 ± 0.08	p = 0.441
Dichotomized IMT (advanced atherosclerotic group(> mean + 1SD)									
CCA	98 (12.7%)	23 (14.5%),	OR = 1.2 (0.7-1.9) p = 0.584	43 (11.7%)	17(22.4%)	OR = 2.1 (1.1-4.1) p = 0.032	55 (13.5%)	6 (7.2%)	OR = 0.5 (0.2-1.2) p = 0.109
Bif	110 (14.2%)	30 (18.9%),	OR = 1.5 (0.9-2.3) p = 0.099	53 (14.5%)	17 (22.4%)	OR = 1.8 (0.9-3.3) p = 0.078	57 (14.0%)	13 (15.7%)	OR = 1.1 (0.6-2.2) p = 0.686
ICA	96 (12.5%)	20 (12.7%),	OR = 1.0 (0.6-1.7) p = 0.940	36 (9.9%)	7 (9.2%)	OR = 0.8 (0.3-1.9) p = 0.588	60 (14.8%)	13 (16.0%)	OR = 1.1 (0.6-2.2) p = 0.724

OR (95% confidence interval) was obtained from logistic regression with adjustment of age, hypertension, diabetes, hyperlipidemia and smoking.

Age-adjusted IMT was presented as mean ± SD

In the overall population, SNP rs702553 was not significant for IMT at any carotid segment. For the dichotomized IMT data in men, the TT genotype had an OR of 2.1 (nominal p = 0.032) for a thicker CCA IMT in comparison to the A allele carriers. However, the p value was not significant after Tukey's ad hoc correction (corrected p = 0.14). The T allele was associated with an increased IMT at Bif (p = 0.078) or ICA (p = 0.588) in men, although the association was not statistically significant. For women, the data was not significant for IMT at any carotid segment.

**Figure 1:**
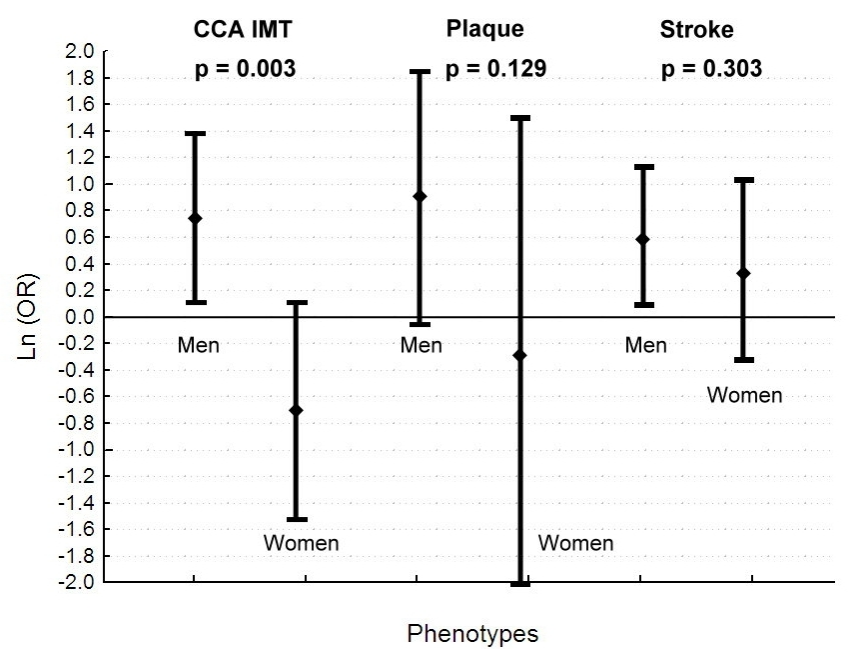
**Odds ratios for men and women between SNP rs702553 and the various phenotypes**. Mean log odds ratios along with the corresponding confidence intervals for men and women are shown. The p value under a Z distribution is used to test the difference of mean ln(OR) between men and women.

We first used regression analysis to evaluate the sex-differential effect of the PDE4D gene. For CCA IMT, the existence of sex-genotype interaction was supported by a p value of 0.009 for the interaction term in the regression model. There was a borderline significance for the sex-genotype interaction (p = 0.062) when plaque index was the phenotype of interest. We further compared the odds ratios in men and women between SNP rs702553 and the various phenotypes (Figure 1). For CCA IMT, the TT genotypes conferred a significantly higher odds ratio in men than in women (OR (95% CI) = 2.16 (1.16-4.02) and 0.5 (0.21-1.16) respectively, p = 0.003). There was no significant difference in the odds ratios for men and women in other phenotypes.

**Figure 2:**
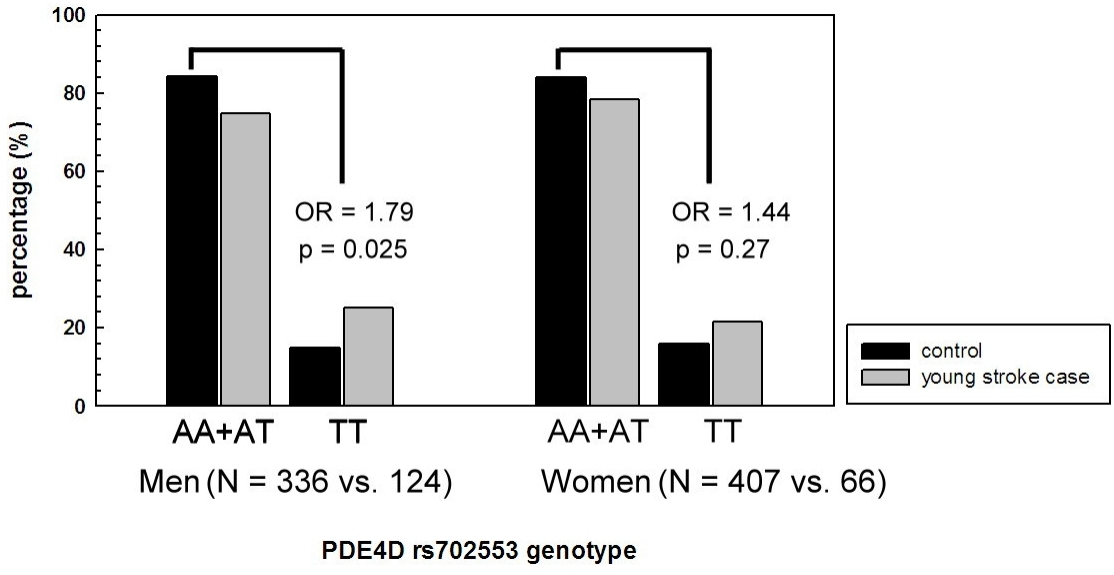
**The association between SNP rs702553 and ischemic stroke in men and women**. The numbers in the parentheses represent the sample size in controls and cases respectively.

The results from carotid atherosclerotic phenotypes were in concordance with the results observed in our previous PDE4D young stroke study [27] where the TT genotype was over-represented in young stroke cases than in the controls (23.9% vs. 15.9%, p = 0.01, Table 1). The TT frequency in the control subjects resembled that in the IMT study population who had no histories of stroke or MI. Compared with the A allele carriers (AA + AT genotypes), the TT genotype conferred an increased risk for stroke in men (OR = 1.79, p = 0.025) but not in women (OR = 1.44, p = 0.27) (Figure 2).

## Discussion

The present study demonstrates a sex-differential effect of the PDE4D gene on CCA IMT and plaque. The TT genotype at SNP rs702553 is associated with an increased risk not only for ischemic stroke but also for the preclinical carotid atherosclerosis. The consistent genetic effect on various atherosclerotic phenotypes suggests that one of the major contributions of the PDE4D gene is to increase atherosclerosis. Our results indicate that the influence of the PDE4D gene begins long before the ischemic infarct, and its genetic effect on stroke is at least partially attributed to the wall thickening and plaque formation in the carotid arteries. In the present study, the pro-atherogenic effect of PDE4D is mainly present in men.

One of the strengths of the present study is using the intermediate phenotypes as proxies of the more complex clinical outcome (i.e. stroke). To our knowledge, there was only one study investigating the PDE4D effect on carotid atherosclerosis [26]. Bevan et al. found a borderline significance for IMT but a negative result for plaque. It should be noted that their study [26] used the arbitrary cut off point of 1.8 mm of wall thickness to define the presence or absence of plaque; whereas we used both the size and the quantity of plaques to calculate the plaque index.

We used both IMT and plaque index to estimate the atherosclerosis severity because they measure different aspects of atherogenesis [32]. IMT mainly represents hypertensive hypertrophy of the vessel walls in response to systemic hypertension and aging [22]. In contrast, plaque probably reflects a later stage of atherosclerosis when lipid infiltration, inflammation, matrix over-production, endothelium dysfunction, and smooth muscle cell proliferation take place [33]. Our results showed that the PDE4D genetic effect is more prominent on plaque index than CCA IMT. It implies that this gene plays a more important role in the pathological process than the physiological changes as IMT. In addition, the modest correlation between plaque index and IMT supports that these two phenotypes represent different aspects of atherosclerotic process. In the present study, the genetic effect of PDE4D is strongest in the CCA IMT, attenuated in the Bif IMT, and completely disappeared in the ICA IMT. Genes with differential effects on different carotid segments have been reported previously [34,35].

One intriguing finding in the present study is the potential sex-differential effect of the PDE4D gene. This sex-genotype interaction has not been reported in the PDE4D gene. However, sex-specific effect has been demonstrated in other genes [36,37]. For examples, the endothelial NO synthase gene (NOS3) affects vascular stiffness in women but not in men [36]. The solute carrier family 2 member 9 (SLC2A9) gene accounts for a larger proportion of uric acid variation in women than in men [37]. Notably, 37.7% of our female subjects were below the age of 50 years. They were still under the anti-atherosclerotic protection of estrogen [38,39], which might also partially explain the lack of PDE4D genetic effect on the female subjects. Joakimsen et al. reported a low plaque prevalence in women younger than fifties and a linear escalation from 10% to 80% at their 4^th ^to 7^th ^decades [39]. Indeed, the fact that only 7 female subjects were in the high-risk group (plaque index ≥ 4) indicates that these women might be too healthy to reveal the PDE4D genetic effect. The small sample size in the high-risk group also explains why a borderline significance was found for the sex-genotype interaction in plaque.

Despite recent studies in the Han Chinese and Korean showed significant associations between the PDE4D gene and certain stroke subtypes [4,40,41], the largest PDE4D association study conducted in the Asian population (including 2847 stroke cases and 4464 controls in Japan) showed a negative result [6]. The main finding of the present study is to demonstrate the PDE4D effect on preclinical carotid atherosclerosis. Therefore, their findings in ischemic stroke may not be comparable to our results in IMT and plaque index.

We selected rs702553 at the PDE4D gene as the candidate locus based on our previous findings in the young stroke study [27]. This 5' SNP is located in a block that has been associated with a reduced expression of the PDE4D7 isoform in stroke patients [3]. In addition, differential expressions of the PDE4D isoforms have been found during the transformation process of vascular smooth muscle cells [42] and monocytes [43]. These studies give a plausible explanation why the intronic SNP rs702553 may modulate IMT progression and plaque formation.

There are several limitations in the present study. First, we only genotyped one SNP at the PDE4D gene and it may not be sufficient to stand for the overall PDE4D genetic effect. Second, the present findings should be interpreted with caution because of the small sample size in subgroup analyses. Yet, the current sample size had a statistical power of 0.51-0.95 to detect a genetic effect of odds ratio 2 with disease prevalence ranged from 10% to 30% and an alpha level of 0.011 http://pngu.mgh.harvard.edu/~purcell/gpc/[44]. Furthermore, we acknowledged that a significant threshold of nominal p less than 0.05 might lead to false positive results when several phenotypes were tested concurrently. We presented both the nominal p values and the Tukey's ad hoc corrected p values. The significance threshold represented a trade-off between avoidance of false positive associations while taking into account that a set of related phenotypes were tested in the present study. We analyzed both continuous and dichotomized IMT data because a certain threshold may be needed in order to detect the atherogenic effect [45]. There was no consensus on the definition of abnormal IMT values and diverse cutoff points were used in different studies [20,46]. We selected mean plus one SD as the cutoff level to dichotomize thick and thin IMT to be comparable to a previous study which used per SD difference in IMT values to estimate the atherosclerotic risks [19]. We also divided the plaque index into three risk groups (index = 0, 1-3, ≥ 4) rather than treating it as a continuous variable for two reasons: (1) plaque index is not normally distributed and the majority of our study subjects (>60%) had no plaque, and (2) the size of plaque could be ambiguous under different insonation angles.

## Conclusions

The present study demonstrates that the rs702553 at the PDE4D gene affects both the IMT progression and plaque formation in the carotid arteries. Men have thicker IMT values, a worse plaque profile, and a higher prevalence of cardiovascular risk factors. Sex may modify the relationships between the PDE4D genes and preclinical atherosclerotic phenotypes. The lack of genetic effect in women warrants further investigation and replication.

## Competing interests

The authors declare that they have no competing interests.

## Authors' contributions

YCL carried out the molecular experiments, participated in statistical analyses and drafted the manuscript. HFL participated in subjects enrollments, carried out the carotid IMT/plaque measurements, and had critical revision of the manuscript. YCG participated in molecular genetic studies and statistical analyses. MLY participated in acquisition of clinical data of study subjects and helped in study design/coordination. CKL participated in study design, data interpretation and study supervision. SHHJ participated in forming the study concept, conceived the study, performed statistical analyses, and drafted the manuscript. All authors read and approved the final manuscript.

## Pre-publication history

The pre-publication history for this paper can be accessed here:

http://www.biomedcentral.com/1471-2350/11/93/prepub
